# Lung Beractant Increases Free Cytosolic Levels of Ca^2+^ in Human Lung Fibroblasts

**DOI:** 10.1371/journal.pone.0134564

**Published:** 2015-07-31

**Authors:** Alejandro Guzmán-Silva, Luis G. Vázquez de Lara, Julián Torres-Jácome, Ajelet Vargaz-Guadarrama, Marycruz Flores-Flores, Elias Pezzat Said, Alfredo Lagunas-Martínez, Criselda Mendoza-Milla, Franco Tanzi, Francesco Moccia, Roberto Berra-Romani

**Affiliations:** 1 Department of Biomedicine, School of Medicine, Benemérita Universidad Autónoma de Puebla, Puebla, Puebla, México; 2 Experimental Medicine Laboratory, School of Medicine, Benemérita Universidad Autónoma de Puebla, Puebla, Puebla, México; 3 Physiology Institute, Benemérita Universidad Autónoma de Puebla, Puebla, Puebla, México; 4 Instituto Nacional de Salud Pública, Centro de Investigación sobre Enfermedades Infecciosas, Cuernavaca, Morelos, México; 5 Instituto Nacional de Enfermedades Respiratorias Ismael Cosío Villegas, México City, México; 6 Laboratory of General Physiology, Department of Biology and Biotechnology ‘‘Lazzaro Spallanzani”, University of Pavia, Pavia, Italy; Cinvestav-IPN, MEXICO

## Abstract

Beractant, a natural surfactant, induces an antifibrogenic phenotype and apoptosis in normal human lung fibroblasts (NHLF). As intracellular Ca^2+^ signalling has been related to programmed cell death, we aimed to assess the effect of beractant on intracellular Ca^2+^ concentration ([Ca^2+^]_i_) in NHLF *in vitro*. Cultured NHLF were loaded with Fura-2 AM (3 μM) and Ca^2+^ signals were recorded by microfluorimetric techniques. Beractant causes a concentration-dependent increase in [Ca^2+^]_i_ with a EC_50_ of 0.82 μg/ml. The application of beractant, at a concentration of 500 μg/ml, which has been shown to exert an apoptotic effect in human fibroblasts, elicited different patterns of Ca^2+^ signals in NHLF: a) a single Ca^2+^ spike which could be followed by b) Ca^2+^ oscillations, c) a sustained Ca^2+^ plateau or d) a sustained plateau overlapped by Ca^2+^ oscillations. The amplitude and pattern of Ca^2+^ transients evoked by beractant were dependent on the resting [Ca^2+^]_i_. Pharmacological manipulation revealed that beractant activates a Ca^2+^ signal through Ca^2+^ release from intracellular stores mediated by phospholipase Cβ (PLCβ), Ca^2+^ release from inositol 1,4,5-trisphosphate receptors (IP_3_Rs) and Ca^2+^ influx via a store-operated pathway. Moreover, beractant-induced Ca^2+^ release was abolished by preventing membrane depolarization upon removal of extracellular Na^+^ and Ca^2+^. Finally, the inhibition of store-operated channels prevented beractant-induced NHLF apoptosis and downregulation of α_1_(I) procollagen expression. Therefore, beractant utilizes SOCE to exert its pro-apoptotic and antifibrinogenic effect on NHLF.

## Introduction

Pulmonary surfactant is a liquid layer covering the alveolar network of mammalian lungs and composed of approximately 90% lipids (mainly phospholipids) and 10% proteins (mainly surfactant-associated proteins or SAPs) [1]. Surfactant accomplishes the biophysical function of reducing surface tension in the alveolar spaces, thereby maintaining alveolar stability and facilitating gas exchange during breathing [2]; in addition, surfactant plays a key role as the front line of defense of pulmonary epithelial cells against inhaled pathogens and toxins [3,4]. However, evidence for other functions has started to emerge. Alterations of the pulmonary surfactant system have been described in infant respiratory distress syndrome (IRDS), adult respiratory distress syndrome (ARDS), obstructive lung diseases, interstitial lung diseases and chronic lung disease [5].

Idiopathic pulmonary fibrosis (IPF) is a chronic, progressive and lethal lung disorder, as patients show a median survival of 3–5 years after diagnosis [6]. IPF is characterized by the accumulation of excessive numbers of fibroblasts and myofibroblasts, exaggerated deposition of extracellular matrix proteins, such as fibrillar collagens, and distortion of normal tissue architecture [7]. The pathogenesis of this disease is still unclear, and the hypothesis of unremitting chronic inflammation as the primary explanation of the pathophysiology of IPF has been challenged by the epithelial injury and activation hypothesis. This hypothesis suggests that chronic noxious stimuli to the alveolar epithelium causes an aberrant activation of the alveolar epithelial cells, as well as abnormalities in the basement membrane integrity, allowing the migration of fibroblasts from interstitium to the alveolar regions of the injured lung, leading to excessive accumulation of extracellular matrix and irreversible loss of the structure of lung parenchyma [8,9]. In accordance with this hypothesis, at some point during the pathogenesis of IPF, fibroblasts come in close contact with the components of the pulmonary surfactant system.

Studies on the effect of surfactant components on non immune cells are scarce. In lung fibroblasts, it has been shown that beractant, an exogenous lung surfactant replacement preparation, downregulates DNA synthesis and inhibits interleukin-1 (IL-1)-stimulated secretion of IL-6 and prostaglandin E2 [10]. Likewise, beractant induces an antifibrotic phenotype in normal human lung fibroblasts (NHLF) by inhibiting the expression of type I collagen, increasing the expression of matrix metalloproteinase (MMP)-1 and promoting fibroblast apoptosis [11]. However, the transduction mechanisms involved in these effects have not been elucidated.

Ca^2+^ signaling is implicated in apoptosis [12], gene expression and phenotypic switch [13], all events related to the effects of beractant on lung fibroblasts. Therefore, we hypothesized that beractant may induce a Ca^2+^ signal in NHLF. An increase in intracellular Ca^2+^ concentration ([Ca^2+^]_i_) can be caused either by Ca^2+^ entry from the extracellular milieu or by Ca^2+^ release from internal storage compartments [14]. The predominant mechanism of intracellular Ca^2+^ mobilization is the inositol 1,4,5-trisphosphate (IP_3_)-induced Ca^2+^ release from the endoplasmic reticulum (ER) [15]. The signal cascade starts typically at the plasma membrane, where the interaction of an extracellular ligand to its cognate tyrosine-kinase or G protein-coupled receptor (TKRs and GPCRs, respectively) activates phospholipase Cγ (PLCγ) or PLCβ. The latter in turn cleaves the membrane phospholipid, phosphatidylinositol 4,5-bisphosphate, into IP_3_ and diacylglycerol [16]. IP_3_ rapidly diffuses to the ER, where it binds to IP_3_ receptors (IP_3_Rs) to mobilize Ca^2+^ into the cytosol, thereby elevating [Ca^2+^]_i_ [17]. While Ca^2+^ release from intracellular Ca^2+^ stores is sometimes insufficient for full activation of cellular processes, extracellular Ca^2+^ entry leads to a more sustained increase in [Ca^2+^]_i_. Ca^2+^ influx is an ubiquitous event that occurs through a number of distinct membrane Ca^2+^-permeable pathways, including voltage-operated, receptor-operated, second messenger-operated and store-operated Ca^2+^ channels (SOCs) [18–20]

It is currently unknown whether beractant alters Ca^2+^ homeostasis in NHLF. Accordingly, we aimed to assess the effect of beractant on [Ca^2+^]_i_ in primary cultures of NHLF, by using conventional imaging microscopy. Our results showed that beractant induces a concentration-dependent Ca^2+^ signal by the concerted activation of PLCβ, Ca^2+^ release from IP_3_Rs and store-operated Ca^2+^ entry (SOCE). The pharmacological blockade of SOCE, in turn, prevented the functional effects of beractant on NHLF, i.e. induction of apoptosis and downregulation of α_1_(I) procollagen expression. Therefore, SOCE is the most likely candidate to mediate the effects of beractant on NHLF.

## Materials and Methods

### Isolation and Purification of Normal Human Lung Fibroblast

Primary NHLF were obtained in our laboratory as previously described [11]. Briefly, NHLF were obtained from kidney donors with brain death and no history of smoking or lung disease and previous signed consent of the family. The protocol was reviewed and approved by the School of Medicine ethics and research committees of the Benemérita Universidad Autónoma de Puebla. After clamping the aorta, a left lung sample was obtained from the lower lobe, one part was processed for histopathology and the other part was minced into small pieces and incubated for 20 minutes with trypsin-EDTA solution and F-12 medium without serum. The digested tissue was gently triturated with a 10 ml pipette. Dissociated cells were filtered through a mesh filter. The filtrate was centrifuged at 200 x g for 10 minutes and the pellet obtained was diluted in F-12 containing 10% fetal bovine serum (FBS) and cultured in T-25 flasks. Cells were grown to a 75% confluence in F-12 medium supplemented with 10% FBS, 100 U/ml of penicillin and 100 μg/ml of streptomycin at 37°C on an atmosphere of 95% O_2_ and 5% CO_2_. Only cells grown from lungs with normal histology were considered for this study. Fibroblasts from passages 5–10 were plated onto coverslips placed in Petri dishes. Cells were allowed to attach to the coverslips for 24 hours, and then incubated for 48 hours in serum free medium.

### [Ca^2+^]_i_ measurements

NHLF attached to the coverslips were washed twice with physiological salt solution (PSS) and loaded with 3 μM Fura-2 acetoxymethyl ester in PSS for 30 min at room temperature. The cells were incubated for 30 min in PSS free of Fura-2. The coverslips were washed and fixed to the bottom of a Petri dish using silicone grease. The Petri dish was mounted onto the stage of an upright epifluorescence Axiolab microscope (Carl Zeiss, Oberkochen, Germany), equipped with a 100-W mercury lamp. A Zeiss X63 Achroplan objective (water-immersion, 2.0 mm working distance, 0.9 numerical aperture) was used to visualize the cells. NHLF were excited alternately at 340 and 380 nm, and the emitted light was detected at 510 nm. A neutral density filter (optical density = 1.0) was coupled to the 380 nm filter to approach the intensity of the 340 nm light. A round diaphragm was used to increase the contrast. The exciting filters were mounted on a filter wheel equipped with a shutter (Lambda 10, Sutter Instrument, Novato, CA, USA). Custom software, working in the LINUX environment, was used to drive the camera (Extended-ISIS Camera, Photonic Science, Millham, UK) and the filter wheel, and to measure and plot on-line the fluorescence from a number of 6–10 rectangular “regions of interest” (ROI) enclosing 6–10 single cells. Each ROI was identified by a number. [Ca^2+^]_i_ was monitored by measuring, for each ROI, the ratio of the mean fluorescence emitted at 510 nm when exciting alternatively at 340 and 380 nm (shortly termed "Ratio (F_340_/F_380_)”. An increase in [Ca^2+^]_i_ causes an increase in the Ratio (F_340_/F_380_). Ratio measurements were performed and plotted on-line every 3 s. Images were stored on the hard disk and converted offline to 340/380 ratio images by ImageJ software (National Institutes of Health, USA, http://rsbweb.nih.gov/ij/). The experiments were performed at room temperature (21–23°C).

### Solutions

PSS had the following composition (in mM): 150 NaCl, 6 KCl, 1.5 CaCl_2_, 1 MgCl_2_, 10 glucose, 10 HEPES. In Ca^2+^-free PSS (0Ca^2+^), Ca^2+^ was substituted with 2 mM NaCl, and 0.5 mM EGTA was added. The osmolality of these solutions was measured with an osmometer (Wescor 5500, Logan, UT, USA). Solutions were titrated to pH 7.4 with NaOH. In Ca^2+^ and Na^+^-free PSS (0Ca^2+^-0Na^+^), extracellular Na^+^ was replaced by an equimolar amount of N-methyl-D-glucamine (NMDG) and the pH was adjusted with HCl.

### Drug Administration

Medium exchange and administration of agonists or other drugs was carried out by first removing the bathing medium (2 ml) by a suction pump and then adding the desired solution. The medium could be substituted quickly without producing artifacts in the fluorescence signal because a small meniscus of liquid remained between the tip of the objective and the cultured NHLF cells.

### Chemicals

Beractant was obtained from Ross Products Division, Abbott Labs (Columbus, OH). Beractant is a sterile, non-pyrogenic pulmonary surfactant. It is a natural bovine lung extract containing 25 mg/mL phospholipids (including 11.0–15.5 mg/mL disaturated phosphatidylcholine), 0.5–1.75 mg/mL triglycerides, 1.4–3.5 mg/mL free fatty acids, and less than 1.0 mg/mL proteins (SP-A and SP-C). It is suspended in 0.9% sodium chloride solution, and heat-sterilized. N-(4-[3,5-bis(trifluoromethyl)-1H-pyrazol-1-yl]phenyl)-4-methyl-1,2,3-thiadiazole-5-carboxamide (BTP-2) was purchased from Calbiochem (La Jolla, CA, USA). Ham’s F-12 medium and FBS were purchased from GIBCO BRL, Life Technologies, Grand Island, NY. All other chemicals were purchased from Sigma-Aldrich.

### Data analysis

For each protocol, data were collected from NHLF isolated from lungs of at least three healthy donors. The amplitude of the peak response was measured as the difference between the ratio at the peak and the mean ratio of 5-min baseline before the peak. Such a difference was considered as a physiological signal when it was > 2 times the SD of the baseline. Ca^2+^ peak and plateau amplitude (ΔR) were normalized to resting fluorescence (Ri) to compare the height of the Ca^2+^ responses to beractant produced by cells displaying different basal fluorescence levels (ΔR/Ri). Mean values are presented together with standard error of the mean and the number “n” of tested cells. Statistical comparisons of peak amplitudes were made by Student’s t-test, p<0.05 was considered significant. As to the plateau phase, the number x of cells responding to the experimental test over the number y of tested cells (x/y) is usually reported. Unless differently stated, tracings shown in the figures are single ROI recordings.

The beractant concentration-response data were fit to an equation of the form:

$$Y=\frac{100}{1+\frac{EC_{50}}{\left[\text{Beractant}\right]}}$$ (1)

where Y is the response (relative to Ca^2+^ transient amplitude), [Beractant] is the beractant concentration, and half-maximal effective concentration (*EC*_*50*_) is the [Beractant] that caused 50% of the response of control.

### RT-qPCR

Real time quantitative polymerase chain reaction was used to measure the expression of collagen in human fibroblasts after 24 hours of incubation. Total cell RNA was extracted with TRIzol reagent (Invitrogen Life Technologies, Grand Island, NY) as per the manufacturer's instructions. RNA was reverse transcribed into cDNA and real-time PCR was performed in the Step One Real-Time PCR System (Applied Biosystems, Foster City, CA) with TaqMan probe labeled with FAM (Hs00164004_m1 for _1-type-I collagen). Gene target levels in each sample were normalized against GAPDH as internal control. Cycling conditions were 2 min at 95°C followed by 40 cycles of 15 s at 95°C and 1 min at 60°C concluding with an infinite loop of refrigeration. All real time PCRs were performed in triplicate at least two times. Results were normalized to human GAPDH according to the delta-delta Ct method (2^-ΔΔCt^).

### Caspase activity assay

The activity of caspases-3 and 7 was measured using the colorimetric Caspase-Glo assay kit according to the manufacturer’s instructions (Promega, Madison, WI). Each well of a 96 well/culture plate contained 10,000 fibroblasts in Ham F12 medium; the plate was incubated at room temperature with 100 μl Caspase-Glo reagent for thirty minutes. A blank reaction was included which only contained cell culture medium without cells. The luminescence of each sample was measured in a plate-reading luminometer (Glomax, Promega, Madison WI).

## Results

### Ca^2+^ response to beractant is heterogeneous in lung fibroblasts

**Fig 1:**
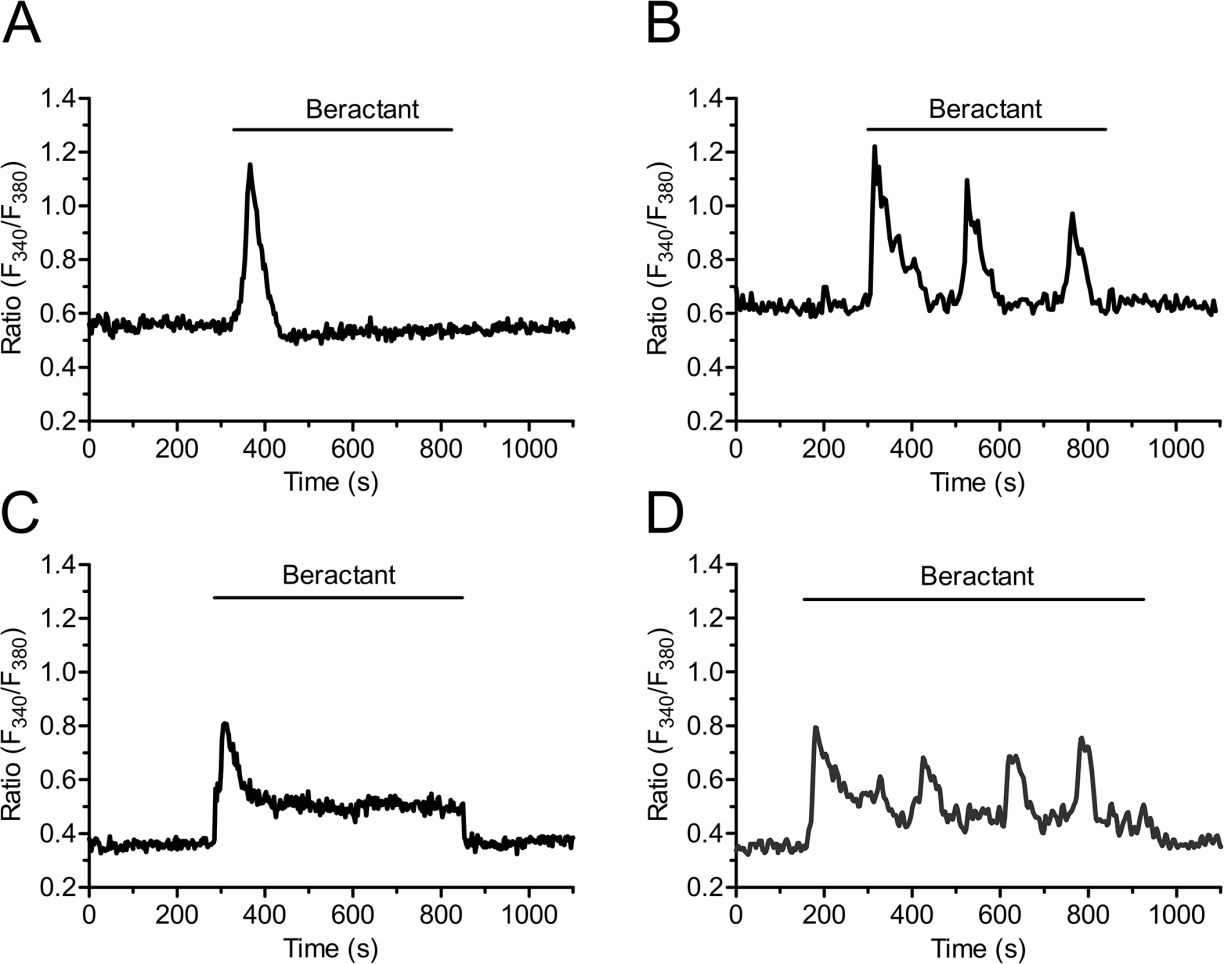
Heterogeneity in the Ca^2+^ response elicited by beractant in NHLF. The application of 500 μg/ml of beractant, elicited different patterns of Ca^2+^ signals in cultured NHLF loaded with Fura-2. The intracellular Ca^2+^ signal consisted in **A)** a rapid Ca^2+^ spike (27.82% of the cells tested) which could be followed by **B)** Ca^2+^ oscillations (13.20%), **C)** sustained plateau (44.37%) or a **D)** plateau overlapped by Ca^2+^ oscillations (14.61%).

In resting, non stimulated NHLF, the application of 500 μg/ml beractant, a concentration which has been shown to exert a nearly maximum apoptotic effect and decrease in collagen accumulation in NHLF [11], caused a Ca^2+^ signal in 96.27% of Fura-2-loaded cells (568 out of 590 cells). Beractant elicited a heterogeneous pattern of Ca^2+^ signals even in cells from the same microscopic field (Fig 1). Accordingly, the intracellular Ca^2+^ signal evoked by beractant consisted in a rapid Ca^2+^ spike (158/568, 27.82%; Fig 1A), that could be followed by Ca^2+^ oscillations (75/568, 13.20%; Fig 1B), a sustained plateau (252/568, 44.37%; Fig 1C), or a plateau overlapped by Ca^2+^ oscillations (plateau + oscillations, 83/568, 14.61%; Fig 1D). Conversely, the IP_3_-synthesizing autacoid ATP (100 μM) induced a biphasic Ca^2+^ response in all the cells analyzed (n = 28) (S1A Fig).

### The pattern and amplitude of the Ca^2+^ response to beractant depends on basal [Ca^2+^]_i_ in quiescent lung fibroblasts

**Fig 2:**
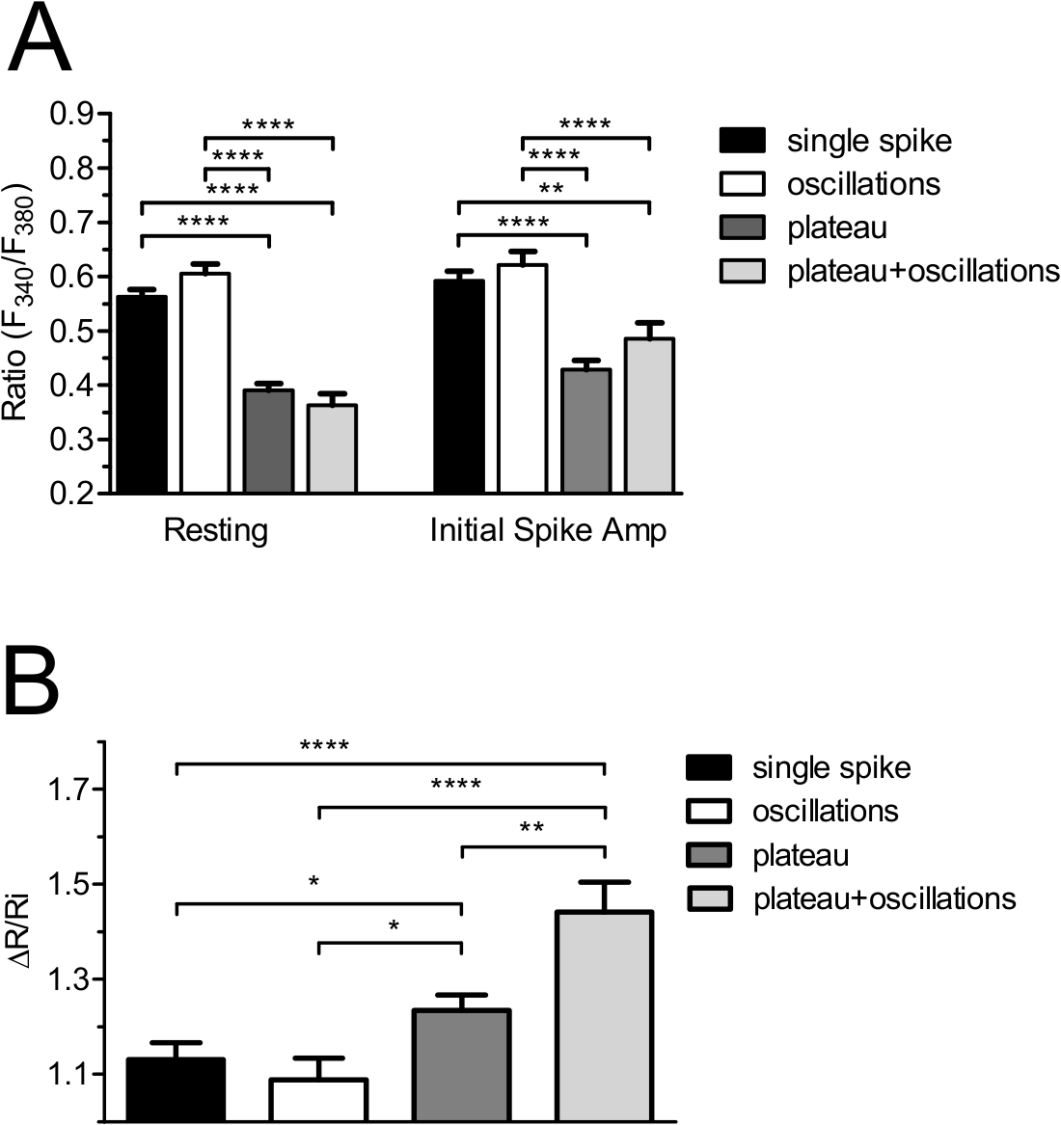
The pattern and initial spike-amplitude of the Ca^2+^ response to beractant depend on resting [Ca^2+^]_i_. **A)** Resting [Ca^2+^]_i_ measured in quiescent, non stimulated Fura-2 loaded NHLF that presented the correspondent Ca^2+^ signal pattern indicated in labels in response to beractant (left). Amplitude of the initial Ca^2+^ spike evoked by beractant (right). **B)** Initial spike amplitude/basal [Ca^2+^]_i_ (ΔR/Ri; ΔR was defined as the difference between the peak [Ca^2+^]_i_ after stimulation and the value of the resting [Ca^2+^]_i_, where Ri is the basal level of R). Results, expressed as means ± SE were analyzed statistically by Student's t test. ** *p* <0.01, **** p <0.0001.

By using digital imaging of Fura-2 fluorescence, we have monitored the [Ca^2+^]_i_ simultaneously in many individual NHLF from the same population. The mean value of basal [Ca^2+^]_i_ measured in all cells studied in the present work was 0.463±0.009 ratio arbitrary units (A.U.) (n = 590). In the majority of cells which had a resting [Ca^2+^]_i_ higher than 0.56 ratio A.U (Fig 2A, left panel), beractant evoked a Ca^2+^ signal which displayed either a single spike (n = 158), or repetitive Ca^2+^ oscillations (n = 75) (such as those described in Fig 1A and 1B, respectively). When the resting [Ca^2+^]_i_ was below 0.4 ratio A.U. (Fig 2A, left panel), beractant evoked Ca^2+^ signals featured by the appearance of a plateau phase (i.e. plateau, n = 252, and plateau + oscillations, n = 83, as shown in Fig 1C and 1D, respectively). In addition, the amplitude of initial Ca^2+^ spike evoked by beractant was seemingly higher in cells that presented high resting [Ca^2+^]_i_ at rest (i.e. single spike and oscillations), as related to those that had levels of basal [Ca^2+^]_i_ lower than 0.4 ratio A.U. (i.e. plateau and plateau + oscillations), as summarized in Fig 2A (right panel). However, when the peak fluorescence was normalized to the resting fluorescence (ΔR/Ri), the magnitude of the initial Ca^2+^ spike was significantly (p<0.05) higher in plateauing cells as compared to single-spiking and oscillating cells (Fig 2B). Overall, these results strongly suggest that resting Ca^2+^ levels influence both the magnitude and the pattern of the Ca^2+^ response to beractant.

### Beractant elicits a concentration-dependent increase in [Ca^2+^]_i_

**Fig 3:**
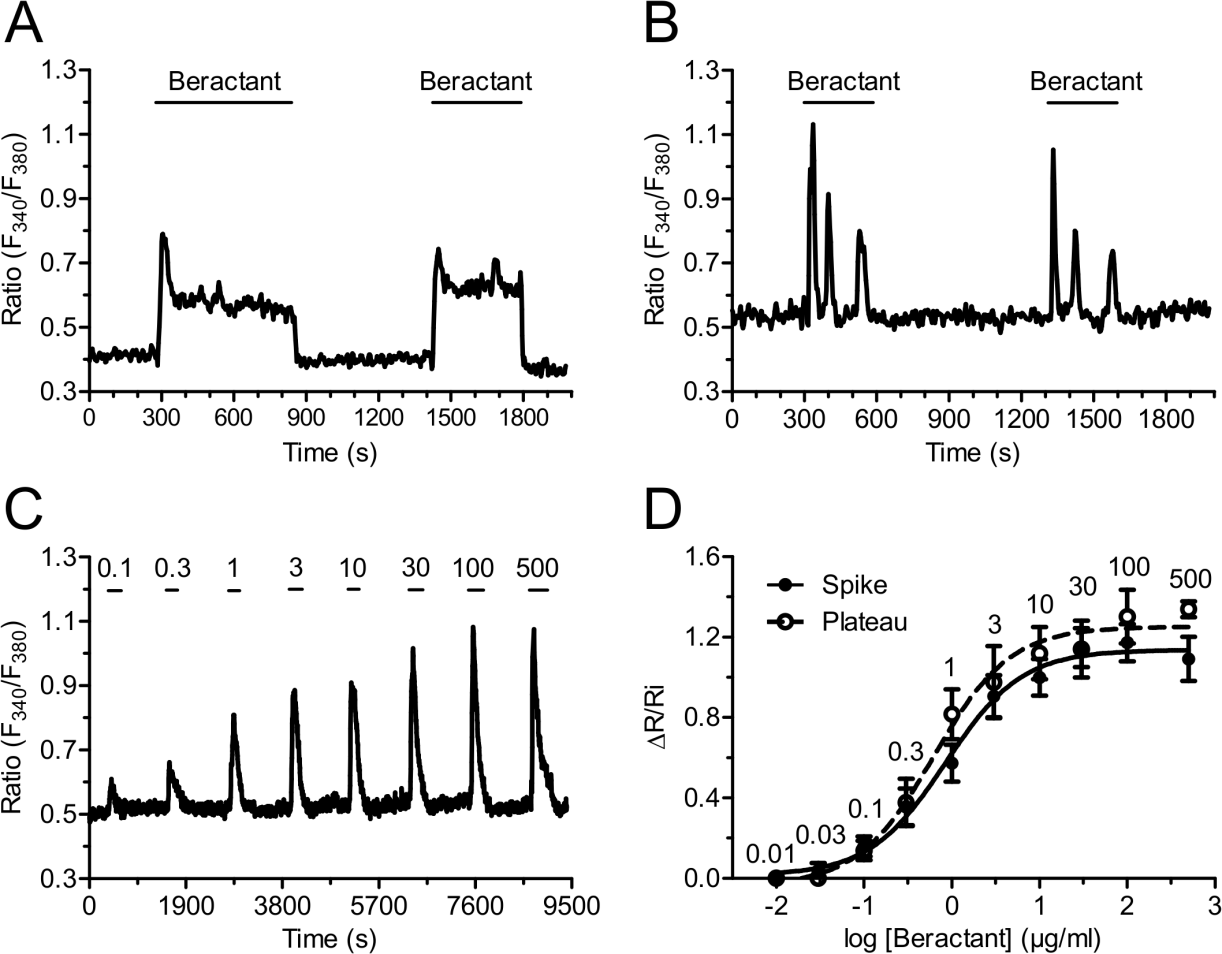
Beractant induces a reversible and concentration-dependent Ca^2+^ signal in NHLF. Ca^2+^ response evoked in a single HNLF cell stimulated repeatedly with the same concentration of beractant (500 μg/ml), a reproducible cell-specific pattern of [Ca^2+^]_i_ signal is observed in: **A)** a cell showing a rapid spike followed by a sustained plateau and **B)** in a cell showing a rapid spike followed by Ca^2+^ oscillations. **C)** A typical trace illustrating the increase in [Ca^2+^]_i_ induced by beractant (0.1–500 μg/ml). **D)** Concentration-response relationship. The ΔR/Ri relationship is plotted against the logarithm of beractant concentration. Data points are means ± SE, n = 7–39 cells. The continuous curves were obtained by fitting the data to Eq 1, which yielded EC_50_ values of 0.8 μg/ml and 0.95 μg/ml for cells exhibiting either a single transient (closed symbols) or a sustained plateau (open symbols), respectively. R^2^ value for the curve fits were 0.9928 and 0.9890, respectively. Numbers into the graphics represent de beractant concentration in μg/ml.

**Fig 4:**
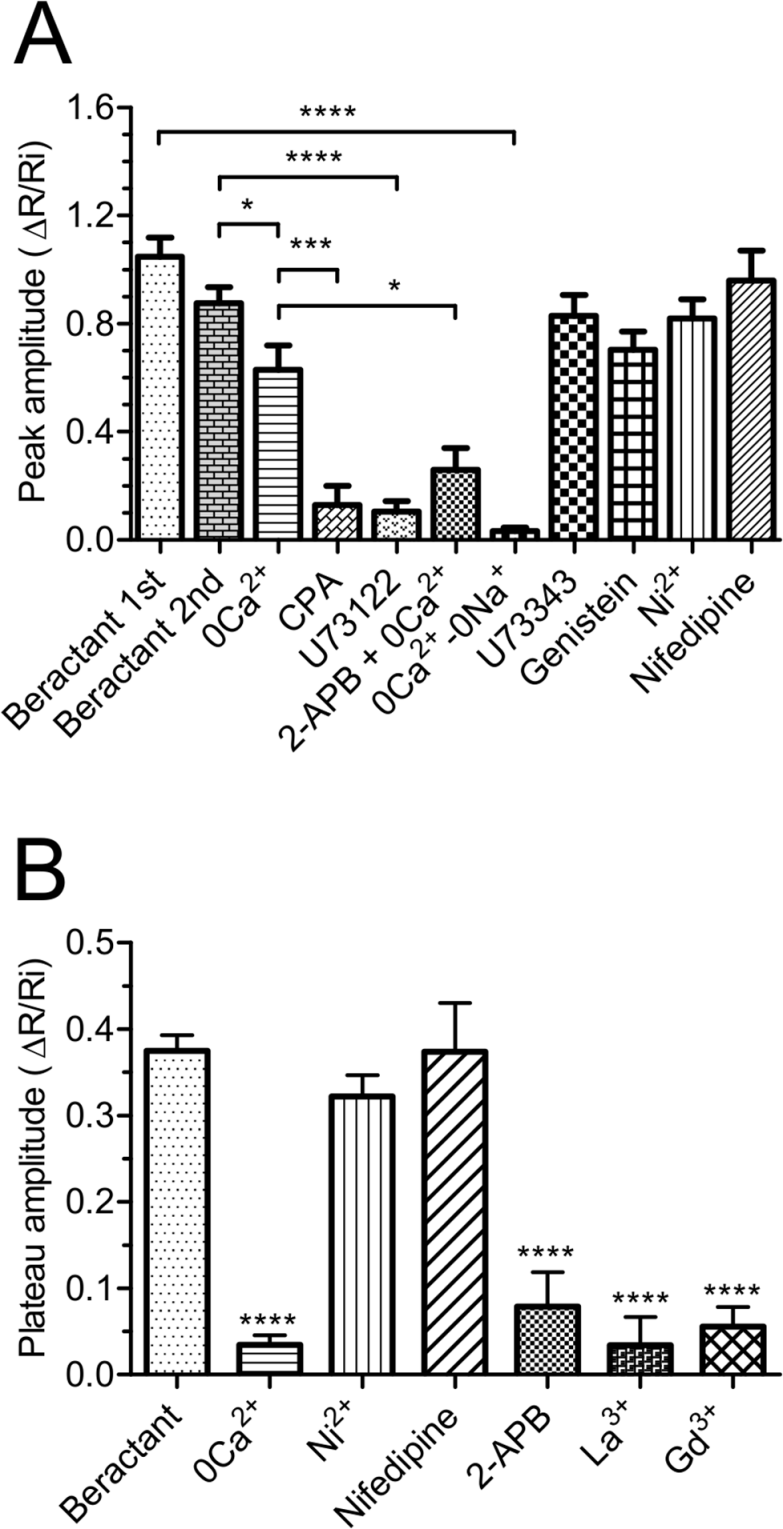
Effect of beractant on the initial Ca^2+^ spike amplitude and plateau phase amplitude in NHLF. **A)** ΔR/Ri for the peak response to beractant in presence of designated drugs. **B)** ΔR/Ri for the plateau phase of beractant-evoked Ca^2+^ increase. See the text for drugs concentrations. Data expressed as means ± SE were analysed statistically by Student’s t-test. * *p* <0.05, *** *p* <0.001,**** p <0.0001.

Beractant effect in NHLF was reversible: the Ca^2+^ signal ceased when the agonist was removed from the bath and a similar Ca^2+^ transient was evoked on beractant restoration. As shown in Fig 3A, the second application of a supramaximal concentration of beractant (500 μg/ml) produced a Ca^2+^ response with similar kinetics and peak amplitude of that evoked by the first application of beractant (n = 55). The same results were obtained when the Ca^2+^ response to beractant consisted in the onset of repetitive Ca^2+^ oscillations (Fig 3B, n = 45). There was a slight reduction in the mean peak amplitude of the second Ca^2+^ transient (13.46%), but the decrease was not statistically significant (Fig 4A, compare Beractant 1st *vs* Beractant 2nd, p > 0.05).

The high reproducibility and lack of desensitization of beractant-induced Ca^2+^ signals enabled us to establish the concentration-response relationship by the repeated administration of the agonist to the same cells. The application of increasing concentrations of beractant (0.03–500 μg/ml) to Fura-2 loaded NHLF produced a concentration-dependent increase in [Ca^2+^]_i_. Fig 3C shows a representative time-course of the Ca^2+^ increases in response to beractant (0.1 to 500 μg/ml) in a NHLF that presented a single spike pattern response (see Fig 1A). Similar results were obtained in NHLF exhibiting the other three patterns of Ca^2+^ response (not shown). The non-cumulative concentration-response curve of beractant-induced elevation in [Ca^2+^]_i_ is depicted in Fig 3D for cells that displayed a single spike (closed symbols) and a plateau response (open symbols). The maximum increase in the peak amplitude was observed at concentrations higher than 100 μg/ml (n = 32 cells), whereas raising beractant concentration up to 500 μg/ml did not significantly augment the height of the response (n = 18 cells). Slight stimulation occurred at 0.1 μg/ml (n = 30 cells), while no effect was detectable at concentrations lower than 0.01 μg/ml (n = 7). The concentration of beractant required to produce a half-maximal response (*EC*_*50*_), calculated by fitting the concentration-response curve as described in Materials and Methods, was 0.82 μg/ml. Notably the R^2^ value for the curve fit was 0.9928 (Fig 3D, closed circles). Similar results were obtained in NHLF cells displaying a long-lasting plateau (Fig 3D, open circles), whose EC_50_ and R^2^ value were 0.95 μg/ml and 0.9890, respectively.

### Beractant triggers the Ca^2+^ response through the PLC/InsP_3_ signaling pathway

**Fig 5:**
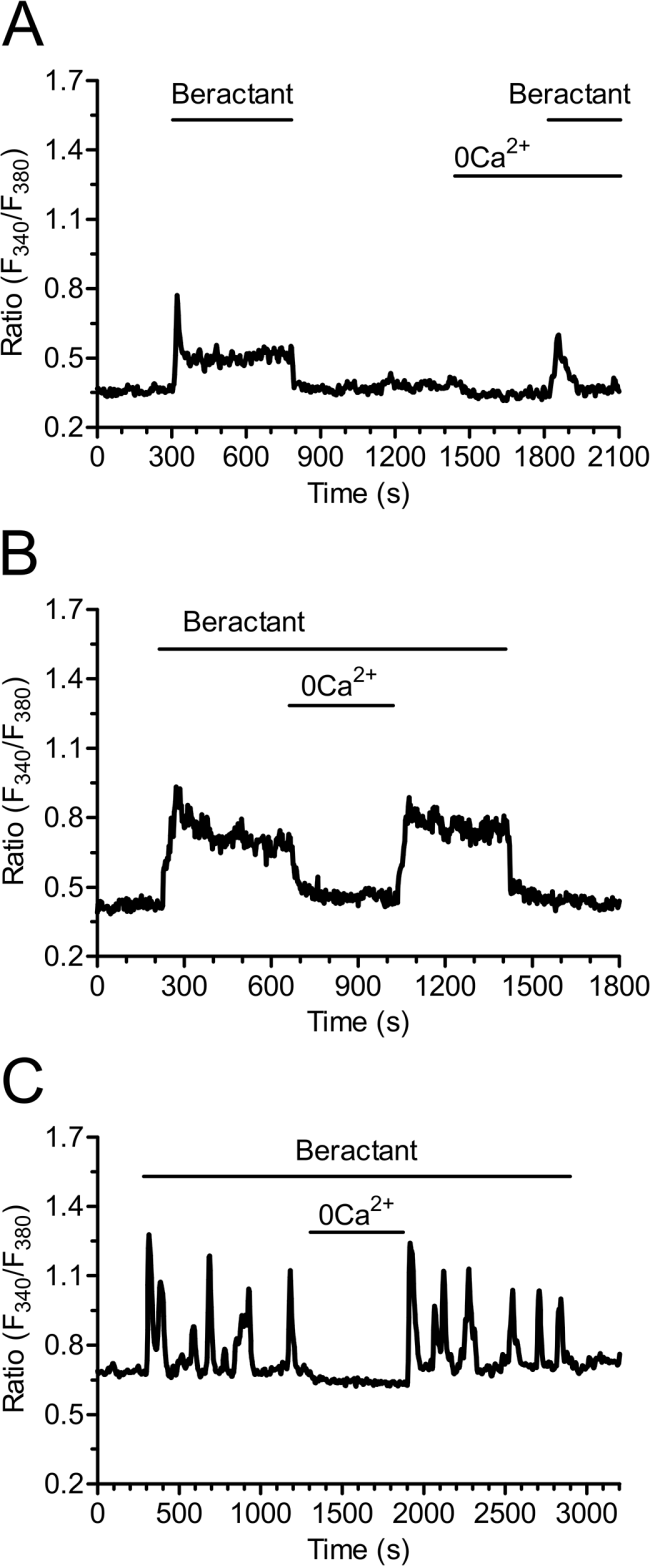
Effect of extracellular Ca^2+^ removal on the Ca^2+^ response to beractant. **A)** Ca^2+^ signal elicited by beractant in the presence and absence of extracellular Ca^2+^ (0Ca^2+^) in the same single cell. Note that the prolonged decay phase did not occur in absence of extracellular Ca^2+^. **B)** Withdrawing extracellular Ca^2+^ (0Ca^2+^) during an established response to beractant (500 μg/ml) immediately interrupted the Ca^2+^ plateau and **C)** repetitive Ca^2+^ oscillations. The Ca^2+^ plateau and Ca^2+^ oscillations resumed upon readmission of external Ca^2+^.

We then sought to dissect the molecular underpinnings of beractant-induced intracellular Ca^2+^ signals. To assess the contribution of intracellular and extracellular Ca^2+^ stores to the Ca^2+^ response to 500 μg/ml beractant, fibroblasts were exposed to the agonist in the absence of external Ca^2+^ (0Ca^2+^) to prevent Ca^2+^ entry through the plasma membrane. Beractant caused an immediate increase in [Ca^2+^]_i_ in the absence of extracellular Ca^2+^ in 48 out of 50 cells, although both the Ca^2+^ oscillations and the plateau phase disappeared (Fig 5A). In addition, the mean amplitude of the initial Ca^2+^ spike observed in Ca^2+^-free solution was significantly reduced by 28.08±10.21% (n = 48; p<0.05) as compared to the Ca^2+^ transient evoked by beractant in presence of extracellular Ca^2+^ (see statistics in Fig 4A: compare 0Ca^2+^ vs Beractant 2nd). These results indicate that the peak response is due to both Ca^2+^ influx and Ca^2+^ release, whereas Ca^2+^ entry sustains both the plateau phase and the following oscillations in [Ca^2+^]_i_. This notion is corroborated by the experiment depicted in Fig 5B, where removal of extracellular Ca^2+^ reversibly inhibited the plateau in 28 of 28 cells (Fig 4B; p< 0.0001, n = 28), and in Fig 5C, which shows the abrupt interruption of beractant-induced repetitive Ca^2+^ spikes in 16 out of 16 cells. Both the plateau phase (Fig 5B) and the Ca^2+^ oscillations (Fig 5C) resumed upon Ca^2+^ restoration to the bath.

**Fig 6:**
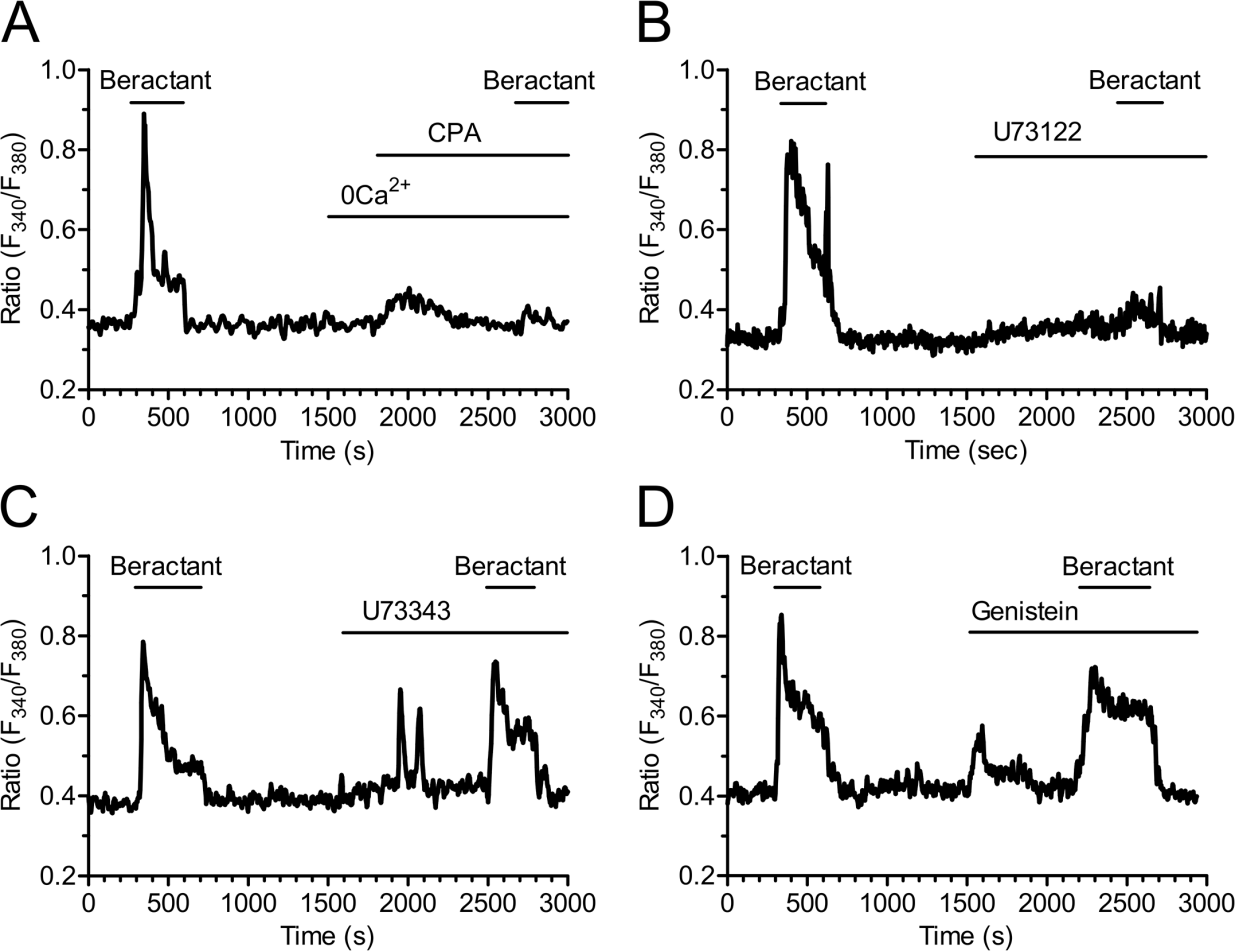
Beractant releases Ca^2+^ from intracellular stores by activating PLC. The initial Ca^2+^ increase evoked by beractant was: **A)** abolished by depletion of intracellular Ca^2+^ stores with CPA (10 μM) in absence of extracellular Ca^2+^ (0Ca^2+^), and **B)** by blockage of PLC activity with U73122 (10 μM), a widely used PLC blocker, **C)** but not by its inactive analogue U73343 (10 μM), **D)** and by genistein (100 μM), a tyrosine kinase inhibitor.

No Ca^2+^ signal was ever observed after depletion of the intracellular Ca^2+^ reservoir by cyclopiazonic acid (CPA, 10 μM) (Fig 6A); CPA is an inhibitor of the ER Ca^2+^-ATPase that prevents Ca^2+^ reuptake into the stores, thus leading to their depletion [20–22]. In Ca^2+^-free solution, CPA evoked a transient increase in [Ca^2+^]_i_ due to passive emptying of the intracellular Ca^2+^ reservoir through ER leak channels and decreased the Ca^2+^ signal elicited by beractant by 79.39% in 16 of 16 cells (Figs 6A and 4A; p< 0.001 n = 16). These findings suggest that the onset of the Ca^2+^ signal evoked by beractant depends on Ca^2+^ mobilization from the intracellular Ca^2+^ pool. Consistently, CPA blocked also the Ca^2+^ response to ATP (n = 10) (S1B Fig).

**Fig 7:**
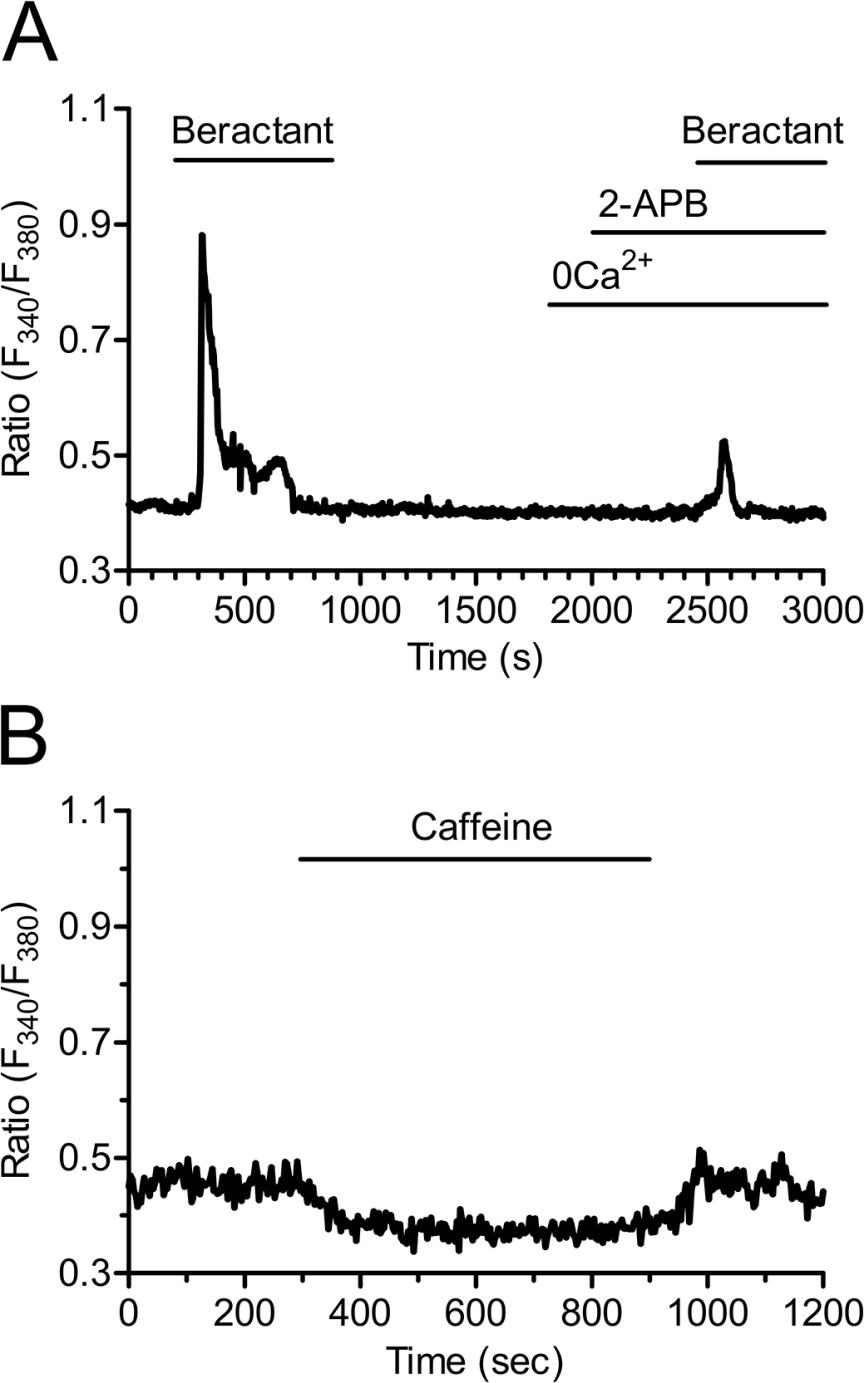
Inositol-1,4,5-trisphosphate receptors (IP_3_Rs) drive the Ca^2+^ response to beractant. **A)** The Ca^2+^ signal elicited by beractant is inhibited in the presence of 2-APB (50 μM), a well known InsP_3_R inhibitor. These experiments were conducted in the absence of extracellular Ca^2+^ (0Ca^2+^) as 2-APB has also been reported to affect SOCs at this concentration. **B)** Caffeine (10 mM), which is a membrane-permeable RyR stimulator, failed to increase [Ca^2+^]_i_ in NHLF.

The involvement of PLC in the transduction pathway leading to beractant-evoked Ca^2+^ signals was studied by preincubating the cells with U73122 (10 μM), a widely employed PLC inhibitor [23–25]. Accordingly, U73122 (10 μM) inhibited the Ca^2+^ response to ATP in 14 out of 16 NHLF (S1C Fig). Cell pretreatment with U73122 caused a significant reduction in the peak amplitude of beractant-evoked Ca^2+^ transient (87.7± 4.25%, n = 19, p<0.0001) (Figs 6B and 4A). Conversely, its inactive structural analogue, U73343 (10 μM), did not significantly affect the Ca^2+^ response to beractant in 19 out of 19 cells (Fig 6C). In the majority of the cells (i.e. 12 out of 19), U73343 (10 μM) exerted little or no effect on basal [Ca^2+^]_i_, but in a fewer cells (7 out of 19 cells) it caused a slow rise in intracellular Ca^2+^ levels accompanied by the development of several Ca^2+^ spikes; however, beractant-induced Ca^2+^ elevation was neither prevented nor affected (Figs 6C and 4A; p> 0.05, n = 19). Taken together, these data suggest that the initiation of the Ca^2+^ signal by beractant requires the activation of PLC and the release of Ca^2+^ from ER stores, presumably through the IP_3_-sensitive Ca^2+^ channels. In order to assess whether PLC activity is triggered following TKR activation, NHLF were preincubated with 100 μM genistein, a widely used TKR inhibitor [25–27]. This maneuver did not prevent or alter the Ca^2+^ response of NHLF to 500 μg/ml beractant (Figs 6D and 4A; n = 8; p>0.05). Genistein reduced the amplitude of the initial Ca^2+^ spike evoked by beractant by 19.47±7.5%, however, no statistically relevant difference was found (p> 0.05) (Fig 4A, compare Genistein *vs* Beractant 2nd). Therefore, PLCβ is the most likely isoform involved in the generation of beractant-induced Ca^2+^ signals. The contribution of IP_3_-dependent signaling was further probed by exposing the cells to beractant in the presence of 2-aminoethoxydiphenyl borate (2-APB; 50 μM), a widely employed inhibitor of IP_3_Rs. These experiments were conducted in the absence of extracellular Ca^2+^ as 2-APB has also been reported to affect SOCs at this concentration [28–30]. Accordingly, this treatment dramatically reduced beractant-induced Ca^2+^ discharge from ER by approximately 58.69% (Figs 7A and 4A, p< 0.05, n = 18). Moreover, caffeine (10 mM), which is a membrane-permeable stimulator of ryanodine receptors (RyRs), failed to increase [Ca^2+^]_i_ in 16 of 16 NHLF tested (Fig 7B). These results, therefore, hint at IP_3_Rs as the main mediators of Ca^2+^ release from ER upon exposition to beractant.

### SOCE sustains the Ca^2+^ response to Beractant

**Fig 8:**
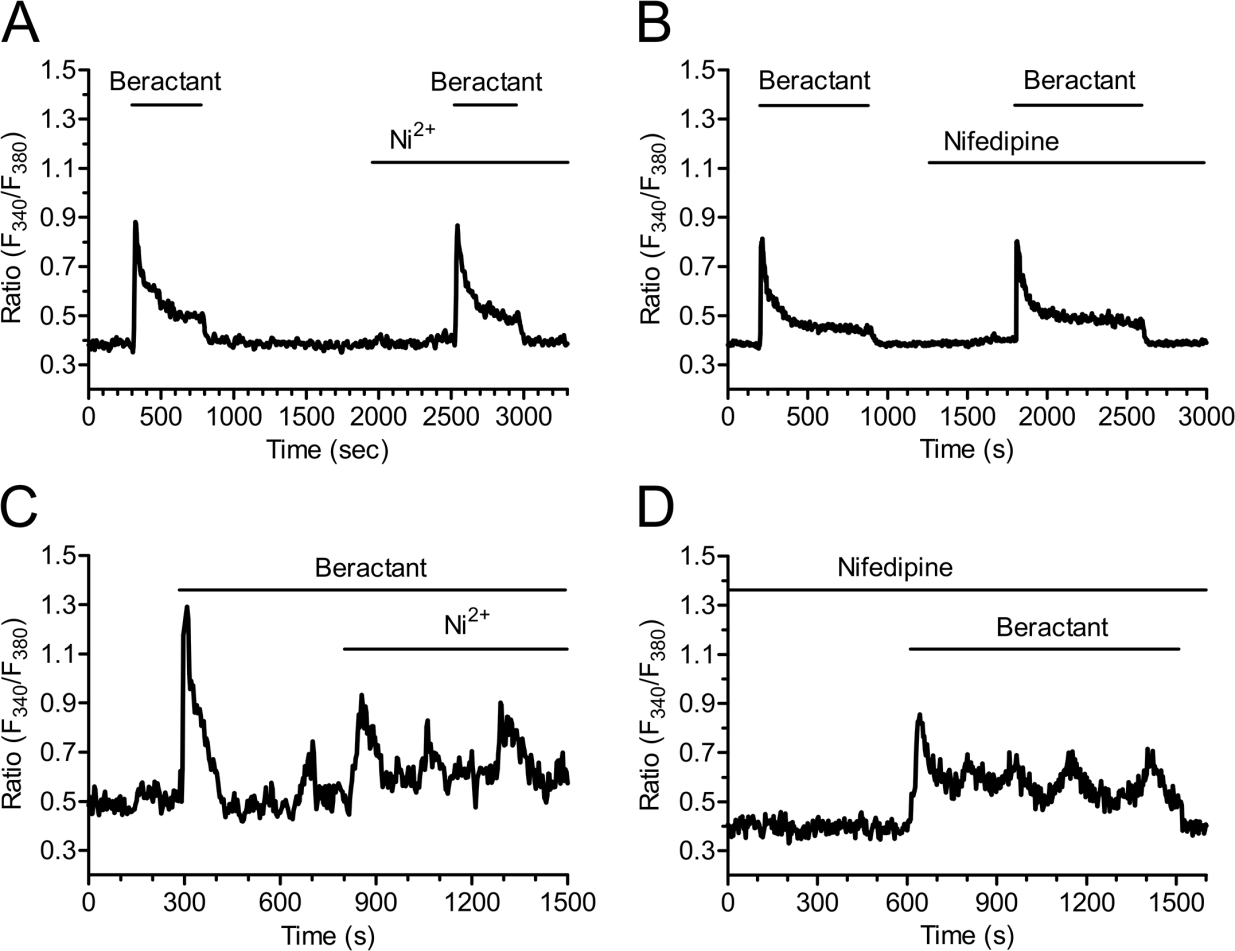
The plateau phase evoked by beractant is not mediated by voltage-operated calcium channels. The plateau phase of beractant-evoked Ca^2+^ signals was not affected **A**) by either Ni^2+^ (100 μM) or **B)** Nifedipine (1 μM). Cells were preincubated for 10 min with nifedipine (1μM) before applying beractant (500 μg/ml). **C)** Nifedipine (1 μM) and **D)** Ni^2+^ (100 μM) did not inhibit beractant-induced Ca^2+^ oscillations.

As previously shown, both the prolonged plateau phase (Fig 5B; n = 48) and the oscillations in [Ca^2+^]_i_ that may follow the initial Ca^2+^ spike triggered by beractant (Fig 5C) do not occur in Ca^2+^-free solution. These findings suggest that Ca^2+^ entry from the extracellular space is essential to sustain the elevation in [Ca^2+^]_i_ over time, whatever its sub-cellular temporal dynamics, i.e. plateau or oscillations. Voltage gated L-type Ca^2+^ channels are the main pathway for Ca^2+^ entry in excitable cells, such as neurons and muscle cells [31]. However, Yang and Huang [32] demonstrated that mouse embryonic fibroblasts express voltage-operated Ca^2+^ channels (VOCC) as well. In order to assess the hypothesis that the sustained Ca^2+^ signal evoked by beractant was mediated by VOCC in NHLF, we probed the effects of Ni^2+^ (100 μM), a non specific blocker of VOCC, and nifedipine (1 μM), which selectively antagonizes L-type VOCC. Neither Ni^2+^ (Figs 8A and 4B; p> 0.05; n = 24) nor nifedipine (Figs 8B and 4B; p> 0.05; n = 30) inhibited the sustained Ca^2+^ response elicited by beractant. All together, these results rule out the contribution of VOCC to the plateau phase that may follow the initial Ca^2+^ response to beractant. Similarly, neither Ni^2+^ (Fig 8C) nor nifedipine (Fig 8D) interfered with beractant-induced intracellular Ca^2+^ oscillations.

**Fig 9:**
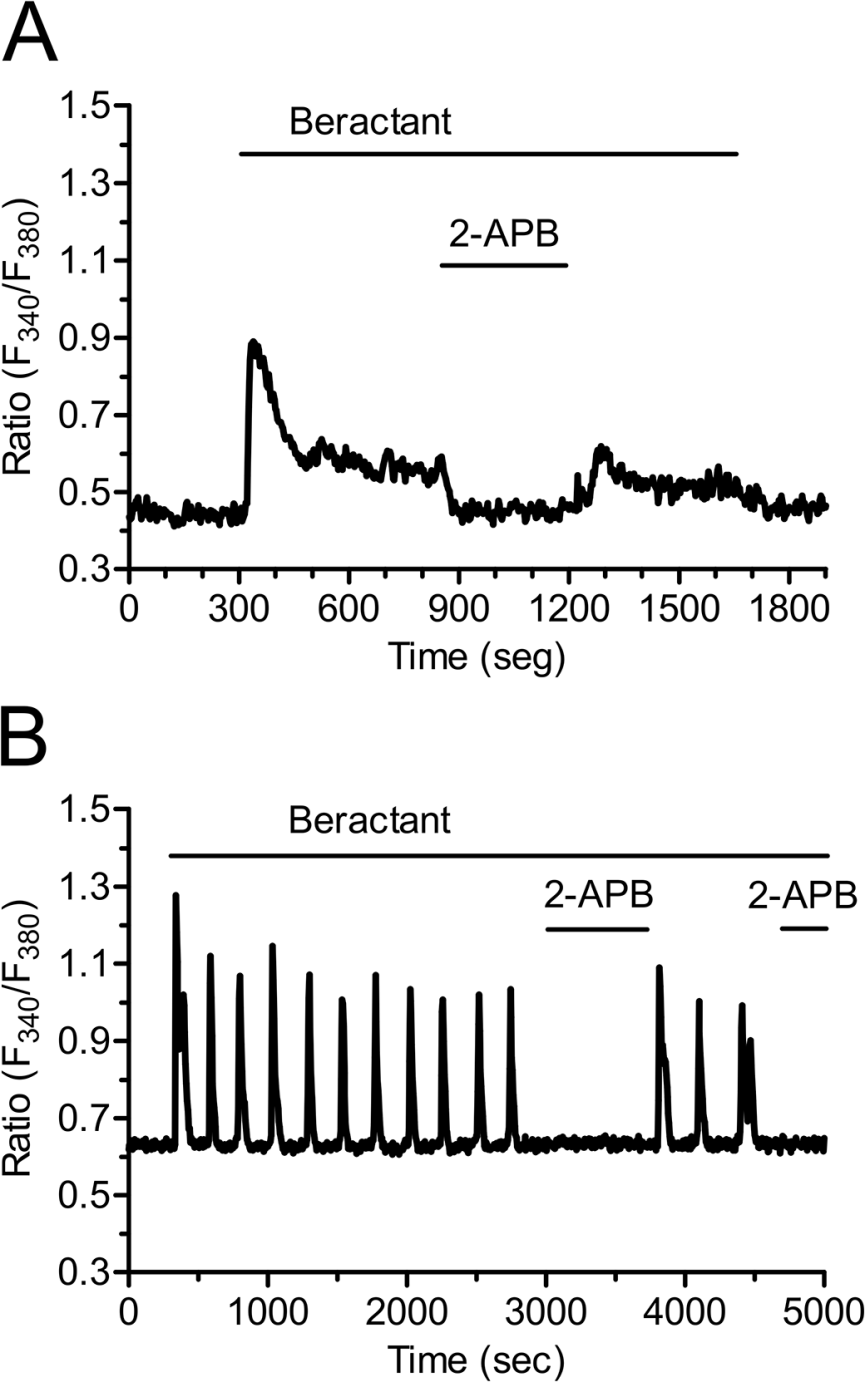
2-APB inhibits beractant-induced Ca^2+^ plateau and Ca^2+^ oscillations in NHLF. 2-APB (50 μM), which may also block SOCs, reversibly inhibited: **A)** the sustained plateau and **B)** the [Ca^2+^]_i_ oscillations evoked by beractant (500 μg/ml) in NHLF.

The most important route for Ca^2+^ inflow into non-excitable cells is represented by SOCE [28,33]. SOCE contribution to beractant-induced Ca^2+^ entry was first assessed by treating the NHLF with 2-APB (50 μM) [34]. In addition to IP_3_Rs, this drug may indeed interfere with SOCE and prevent Ca^2+^ influx in the presence of extracellular Ca^2+^ [29,35]. 2-APB reduced by 79% (p<0.001) the amplitude of the Ca^2+^ plateau in 18 of 22 cell tested (Figs 9A and 4B, p< 0.0001, n = 22). Likewise, 2-APB (50 μM) reversibly abolished beractant-induced oscillations in [Ca^2+^]_i_ in 10 out of 10 cells (Fig 9B).

**Fig 10:**
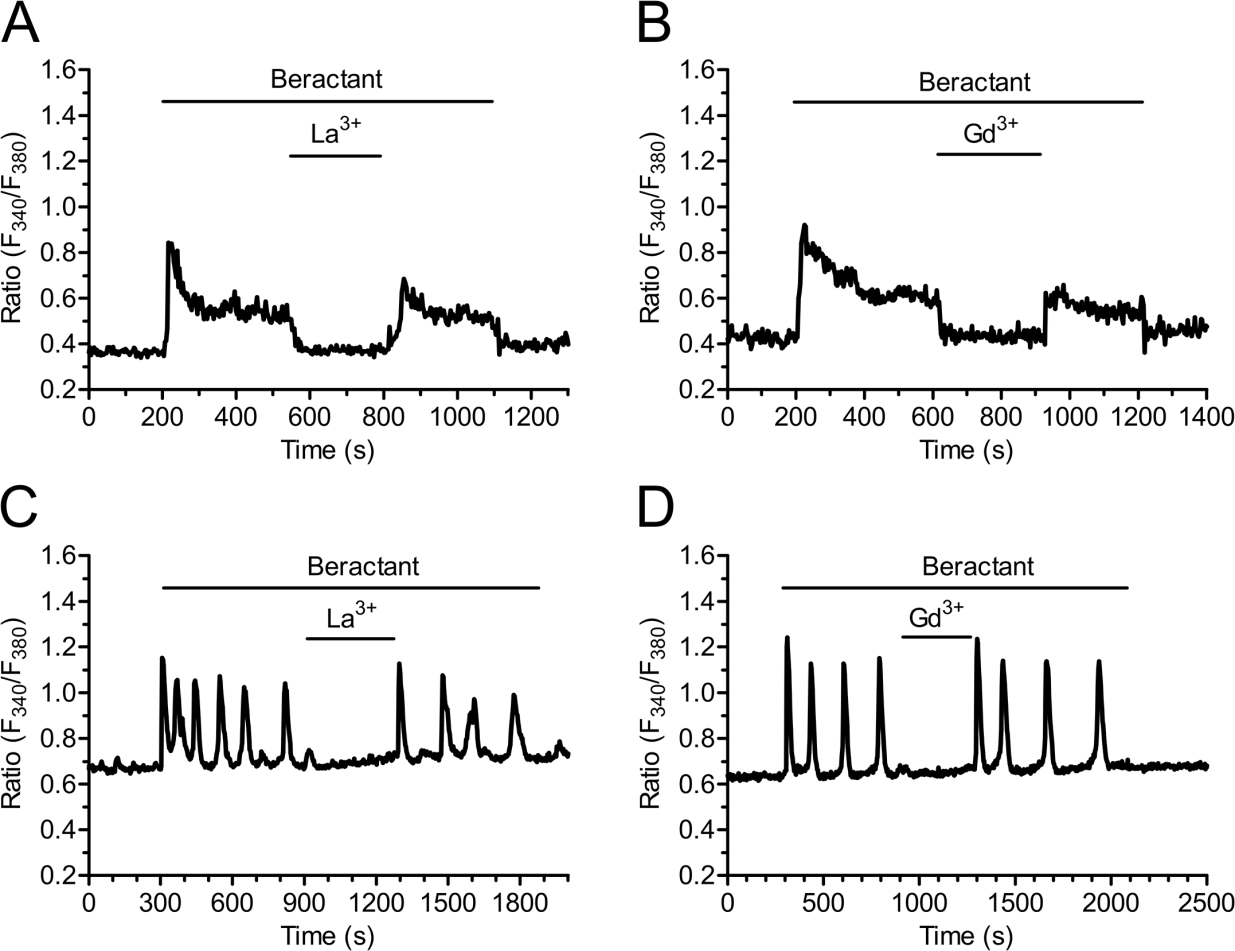
La^3+^ and Gd^3+^ block beractant-induced Ca^2+^ plateau and Ca^2+^ oscillations in NHLF. A) Addition of La^3+^ (10 μM) and B) Gd^3+^ (10 μM) reversibly inhibited the sustained plateau phase of the Ca^2+^ signal induced by beractant (500 μg/ml). C) Application of La^3+^ (10 μM) and D) Gd^3+^ (10 μM) reversibly inhibited beractant-elicited Ca^2+^ oscillations (500 μg/ml).

As shown by the experiments conducted in the absence of external Ca^2+^, these results might be explained by the combinatorial inhibition of IP_3_Rs and SOCE. As a consequence, we exploited three additional well known inhibitors of SOCs, namely the pyrazole derivative, BTP-2, and the trivalent cations, La^3+^ and Gd^3+^ [28,29,36,37]. Unfortunately, our preliminary experiments revealed that 20 μM BTP-2 induced a robust increase in [Ca^2+^]_i_ in 21 of 21 NHLF cells and could not be utilized further (S2 Fig). When applied at concentrations ranging from 1 up to 10 μM, lanthanides are rather selective towards SOCs and do not affect either receptor- or second messenger-operated Ca^2+^ channels [28,29,37]. As illustrated in Fig 10, both La^3+^ (10 μM) and Gd^3+^ (10 μM) reversibly inhibited the plateau phase (Figs 10A and 4B, p<0.0001, n = 18 and Figs 10B and 4B, p<0.0001, n = 23, respectively), as well as the repetitive oscillations in [Ca^2+^]_i_, that followed the initial Ca^2+^ peak induced by beractant (Fig 10C and 10D, respectively). Overall, these results strongly suggest that SOCE maintains the sustained component of the Ca^2+^ response to beractant in NHLF.

### The Ca^2+^ response to beractant is not mimicked by phospholipids, but requires membrane depolarization

**Fig 11:**
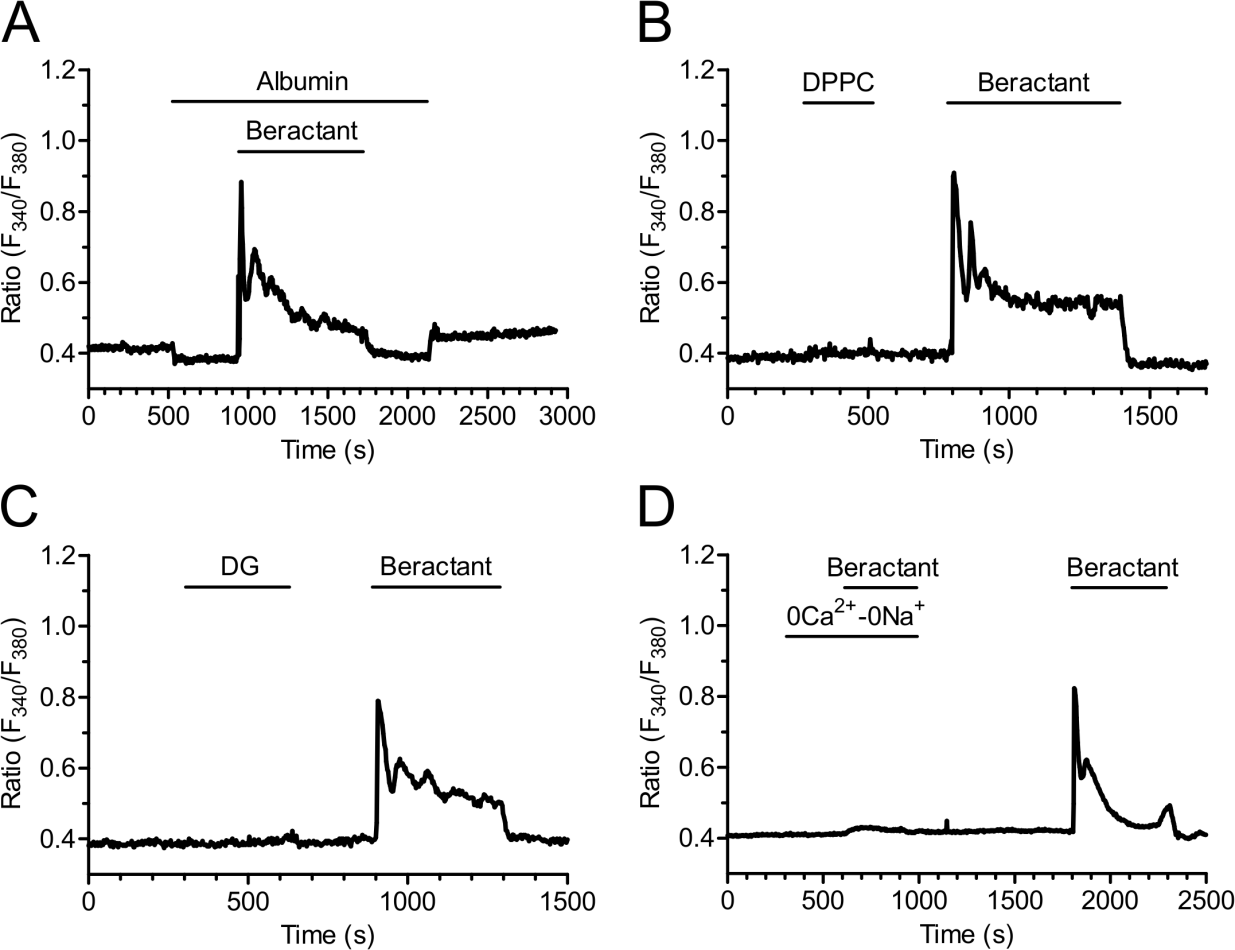
The Ca^2+^ response to beractant is not mimicked by albumin, (DPPC) and diacylglycerol, but is inhibited by preventing membrane depolarization. **A)** Albumin, **B)** dipalmitoylphosphatidylcholine (DPPC) (200 μg/ml) and **C)** diacylglucerol (DG) (50 μg/ml) did not evoke any detectable increase in [Ca^2+^]_i_ in NHLF. **D)** the Ca^2+^ response to beractant (500 μg/ml) was abrogated by replacing extracellular Na^+^ with an equimolar amount of NMDG in the absence of external Ca^2+^ (0Ca^2+^-0Na^+^).

In order to assess which of the single components of beractant trigger the Ca^2+^ response and how they are related to PLCβ activation, we first probed the effect of albumin, dipalmitoylphosphatidylcholine (DPPC) and diacylglycerol (DG). Albumin is a protein which is not associated to lung surfactant, and failed to evoke any Ca^2+^ signal in beractant-responsive NHLF (Fig 11A, n = 18). Actually, albumin caused a slight decrease in basal Fura-2 fluorescence, but this did not prevent beractant from elevating [Ca^2+^]_i_ (Fig 11A). Similarly, DPPC (200 μg/ml) and DG (50 μg/ml), which are two phospholipid constituents of beractant, did not elicit any increase in [Ca^2+^]_i_ (Fig 11B and 11C, n = 15 and 18, respectively). Overall, these findings strongly suggest that the Ca^2+^ response to beractant is mediated by SAPs. More specifically, beractant contains SAP-B and SAP-C, which were recently shown to bring about Ca^2+^ signals through the insertion of monovalent cation channels on the plasma membrane. The resulting depolarization leads to IP_3_-dependent Ca^2+^ release by a yet to be discovered mechanism [38,39]. Therefore, we then analyzed the triggering mechanism of beractant-induced Ca^2+^ release by stimulating NHLF in the absence of external Na^+^ and Ca^2+^ to prevent membrane depolarization. This procedure reversibly abolished beractant-induced increase in [Ca^2+^]_i_ (Fig 11D).

### SOCE inhibitors prevent beractant-induced apoptosis and collagen expression downregulation

**Fig 12:**
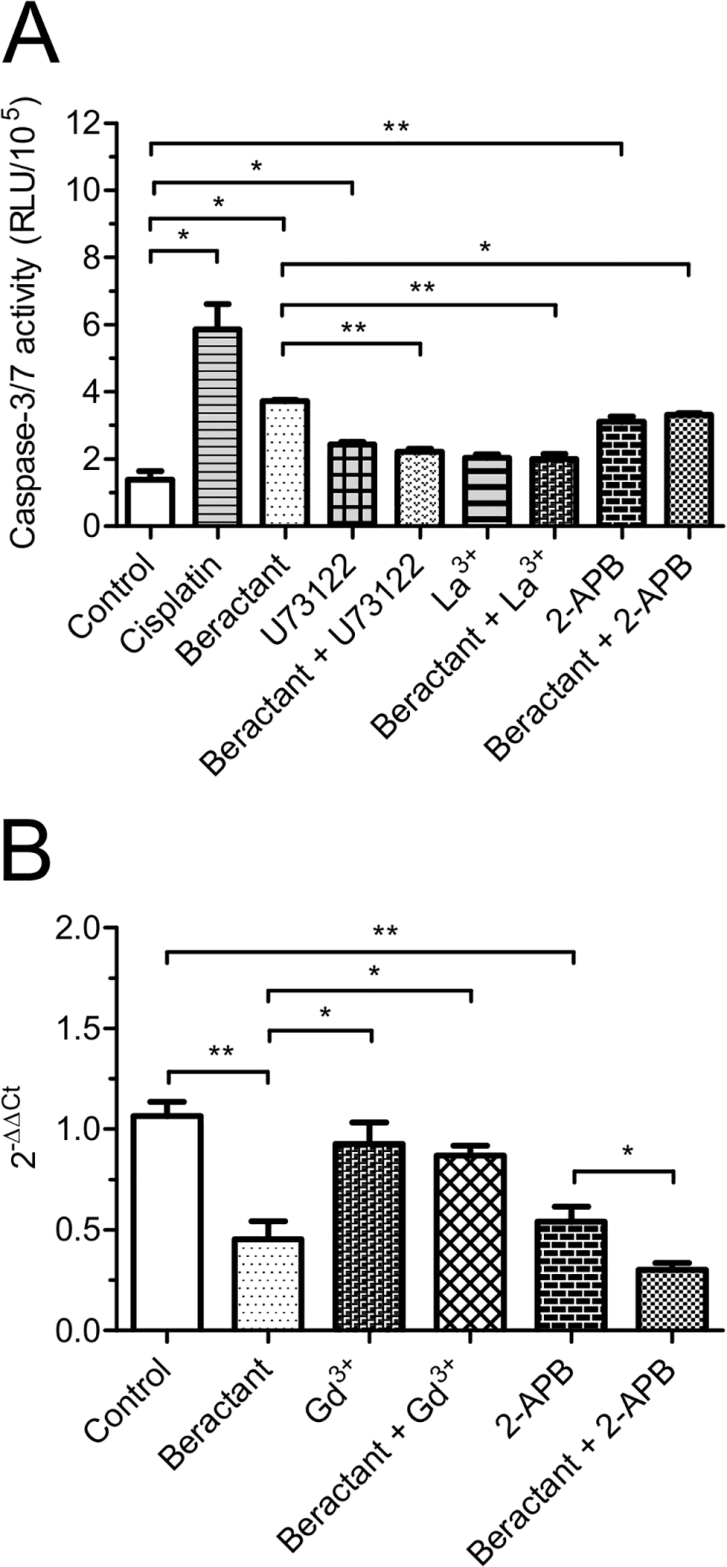
Effect of SOCE inhibitors on Beractant effect in apoptosis and collagen expression. NHLF were incubated for 24 hours with either SOCE inhibitors alone (U73122, 10μM; La^3+^, 10μM; Gd^3+^, 10μM or 2-APB, 50 μM) or in combination with beractant 500 μg/ml in serum-free medium. **A)** Caspase 3 and 7 activity, the Caspase-Glo assay kit (Promega, Madison, WI) was used to measure the executioner caspases 3 and 7. Cisplatin 20 μM was used as positive control. Despite the fact that U73122 and 2-APB exerted a modest, albeit significant, pro-apoptotic effect, they blocked beractant-induced apoptosis. **B)** Collagen expression. RT-qPCR was used to measure the expression of collagen. All real time PCRs were performed in triplicate at least two times. Results were normalized to human GAPDH using the delta-delta Ct method (2^-ΔΔCt^). Results are expressed as means ± SE. ANOVA was used with *a priory* comparisons of selected pairs.

Finally, we investigated the role of intracellular Ca^2+^ signals in beractant-induced NHLF apoptosis and collagen expression. Pre-incubating the cells for 24 hrs with U73122 (10 μM), La^3+^ (10 μM) or 2-APB (50 μM) prevented beractant-induced apoptosis, as evaluated by monitoring caspase 3 and 7 activity (Fig 12A; p < 0.05). Collectively, these data indicate that SOCE promotes the pro-apoptotic effect of beractant in NHLF. Likewise, pre-treating the cells with Gd^3+^ (10 μM, 24 hrs) prevented beractant from downregulating α_1_(I) procollagen gene expression (Fig 12B). Unfortunately, 2-APB (50 μM) caused a reduction in α_1_(I) procollagen gene expression *per se* (Fig 12B) and did not interfere with the action of beractant. These data, however, strongly suggests that SOCE contributes also to reduce beractant-dependent α_1_(I) procollagen transcript.

## Discussion

Restoration of surfactant activity has been introduced in the routine care of patients affected by respiratory distress syndrome [4], and might be a suitable tool to adverse intraluminal fibrosis in IPF and other interstitial lung diseases. Beractant is a natural bovine extract enriched with phospholipids, neutral lipids, fatty acids, and the hydrophobic proteins SP-B and SP-C, and is widely employed in clinical practice, albeit the underlying signal transduction mechanisms are far from being fully elucidated. Importantly, it shows anti-inflammatory and anti-fibrotic properties [10,11]. Ca^2+^ signaling regulates a myriad of cellular processes, including those elicited by beractant in fibroblasts, i.e. DNA replication, gene expression, apoptosis, and differentiation [13,33,40]. In this context, our results provide the first evidence that beractant elicits an heterogeneous increase in [Ca^2+^]_i_ in NHLF, which might be involved in its functional effect on these cells.

Beractant evoked a complex pattern of elevations in [Ca^2+^]_i_ in neighboring NHLF, which displayed at least 4 types of responses upon agonist stimulation: 1) a rapid Ca^2+^ spike which quickly decayed to the baseline; 2) a biphasic Ca^2+^ signal, which comprised an initial Ca^2+^ spike followed by a prolonged plateau phase of intermediate amplitude; 3) repetitive oscillations in [Ca^2+^]_i_ and 4) a biphasic elevation in [Ca^2+^]_i_ featured by the superimposition of Ca^2+^ oscillations on the plateau phase. Studies of the Ca^2+^ responses of a wide variety of cell types at the single cell level have consistently revealed cell-to-cell heterogeneity [23,25,41–44]. Cell cycle heterogeneity as an explanation of the variability in the Ca^2+^ response to beractant is very unlikely. First, all our experiments were carried on in serum-starved NHLF for 48 hours, which arrests cell cycle in G_o_ phase [45]. Second, in spite that cell cycle asynchrony has been proposed as a source of variability in Ca^2+^ signaling, existing data do not support this idea. Thus, synchronization of cultured human foreskin fibroblasts failed to prevent the variety of patterns in [Ca^2+^]_i_ elevations elicited by bradykinin [46]. Similarly, Ambler and Cols [43] showed that synchronized cycling BC3H-1 cells responded asynchronously to histamine stimulation. Finally, serum-starved rat cardiac coronary microvascular endothelial cells still displayed a heterogeneous Ca^2+^ response to EGF [41]. Collectively, these considerations lead us to conclude that individual NHLF produce asynchronous changes in [Ca^2+^]_i_ when exposed to beractant and that this is not due to cell heterogeneity in the cell cycle.

Recently, Ishida and coworkers (2014) showed that cell-to-cell variability in the pattern of Ca^2+^ signals in histamine-stimulated in HeLa cells is due to heterogeneity in the process of IP_3_ production. Modulation of IP_3_ dynamics by knockdown or overexpression of PLCβ1 and PLCβ4 resulted in specific changes in the characteristics of Ca^2+^ signals within the range of the cell-to-cell variability found in wild-type cell populations [44]. Moreover, the cell-specific pattern of beractant–induced increase in [Ca^2+^]_i_ in NHLF might invoke single-cell heterogeneity regarding membrane receptors or elements of the phosphoinositide signaling pathways, such as PLCβ, IP_3_Rs, and SOCE (see below), as recently suggested in [23,47]. An additional, albeit not mutually exclusive, explanation for the cell-to-cell variability observed in beractant-stimulated NHLF resides in their resting Ca^2+^ levels: The basal [Ca^2+^]_i_ is higher in cells displaying either a single Ca^2+^ spike or discrete Ca^2+^ oscillations as compared to those experiencing the plateau phase, with or without the superimposition of sinusoidal oscillations. Similar results were found by Toescu and coworkers [48], who reported about acetylcholine (Ach)-induced intracellular Ca^2+^ waves in cultured mouse pancreatic acinar cells. When the basal Ca^2+^ levels were lower than a threshold concentration of 150 nM, ACh always evoked high frequency short-lasting Ca^2+^ spikes, whereas it elicited less frequent, long-lasting Ca^2+^ transients at [Ca^2+^]_i_ > 150 nM [48]. Future work is required to ascertain how the basal Ca^2+^ concentration impacts on beractant-induced Ca^2+^ signals. Nevertheless, it is possible to conclude that the pattern of the Ca^2+^ response in NHLF is specific to each cell: when a given NHLF is repeatedly stimulated with the same concentration of beractant, a reproducible and cell-specific pattern of [Ca^2+^]_i_ signal, the so-called Ca^2+^ fingerprint [42], occurs (see, for instance, Fig 3A and 3B).

The EC_50_ of beractant-induced elevation in [Ca^2+^]_i_ is equal to 0.82 μg/ml, while its maximal effect was achieved at 100–500 μg/ml. Likewise, in NHLF, beractant was found to induce apoptosis and reduce collagen deposition at 500 μg/ml [11], whereas it interfered with DNA synthesis and secondary inflammatory mediator production at 500–1000 μg/ml [10]. Moreover, beractant was shown to insert plasmalemmal cation monovalent channels and trigger Ca^2+^ release in human neutrophils in the same concentration range [38,39]. Therefore, we believe that the concentrations of beractant employed in the present investigation are very close to those established by other authors. The following pieces of evidence indicate that beractant-elicited intracellular Ca^2+^ signals in NHLF are patterned by the coordinated interplay between IP_3_-dependent Ca^2+^ release and SOCE. First, the increase in [Ca^2+^]_i_ is prevented by U73122, a widely employed PLC inhibitor, while it is unaffected by its structural analogue, U73343. genistein, a broad spectrum protein tyrosin kinase inhibitor, did not interfere with the onset of the Ca^2+^ response to beractant. Therefore, PLC activity is likely to be induced by the activation of GPCRs and to involve the β-isoform. Second, beractant-induced Ca^2+^ signals are prevented by 2-APB, a membrane-permeable blocker of InsP_3_-dependent Ca^2+^ release in the absence of external Ca^2+^. Third, no Ca^2+^ signal could be detected in response to caffeine, which stimulates endogenous RyRs by sensitizing them to resting Ca^2+^ levels [49]. Fourth, Ni^2+^ and nifedipine, two established VOCC blockers, did not affect beractant-induced increase in [Ca^2+^]_i_. Fifth, lanthanides reversibly abrogated the sustained component of the Ca^2+^ response to beractant, by interrupting both the prolonged plateau phase and the repetitive Ca^2+^ oscillations. This effect was observed when both La^3+^ and Gd^3+^ were applied at 10 μM, a concentration which selectively hinders SOCs [28,37,50]. BTP-2, another well known SOCE inhibitor, could not be probed in the present study due to its ability to increase [Ca^2+^]_i_ in NHLF. These findings strongly indicate that, while the single Ca^2+^ transients exclusively derive from IP_3_-dependent Ca^2+^ mobilization, the sustained response involve Ca^2+^ entry through plasmalemmal SOCs. This concept is corroborated by the finding that, when 2-APB is administrated in the presence of extracellular Ca^2+^ to interfere with IP_3_Rs and SOCE, both the prolonged plateau phase and the repetitive Ca^2+^ spikes rapidly run down. According to the most popular models proposed to describe intracellular Ca^2+^ oscillations [28,51,52], SOCE refills the intracellular Ca^2+^ stores during maintained stimulation and provides IP_3_Rs with a sufficient amount of intraluminal Ca^2+^ to sustain their spiking activity. In this scenario, however, Ca^2+^ oscillations do not cease immediately after the removal of extracellular Ca^2+^, but persist for some time in the absence of Ca^2+^ entry. Conversely, beractant-induced Ca^2+^ transients are instantaneously inhibited by perfusing the cells with a Ca^2+^-deficient solution, whereas they quickly resume on Ca^2+^ restoration to the bath. This observation might be explained by a role for Ca^2+^ entry in governing IP_3_-dependent Ca^2+^ release. Environmental Ca^2+^ controls IP_3_-mediated Ca^2+^ mobilization, whereas surrounding Ca^2+^ stimulates or inhibits IP_3_ gating at [Ca^2+^] lower and higher than 10 μM, respectively [53]. The immediate interruption of Ca^2+^ oscillations in 0Ca^2+^ suggests that SOCE is required to achieve adequate levels of stimulating Ca^2+^ nearby intracellular IP_3_Rs, as observed in spiking HeLa cells stimulated with histamine [54] and exocrine epithelial cells challenged with carbachol [55]. The molecular structure of SOCE may vary depending on the cell type [34]. This pathway is mediated by the interaction between the ER Ca^2+^ sensor, Stim1, and the Ca^2+^-permeable channels, Orai1 and TRPC1, in normal rat kidney fibroblasts [56]. Nevertheless, Orai1 and TRPC1 have reported to serve as independent SOCs, each activated by Stim1, in human submandibular gland cells [57]. Consistent with these data, Stim1, Orai1 and TRPC1 have been reported in human cardiac fibroblasts [58]; however, the molecular composition of SOCE in NHLF is yet to be elucidated and will require further work.

Our results are consistent with previous investigations, which demonstrated the influence of pulmonary surfactant on intracellular Ca^2+^ homeostasis in both human neutrophils [38] and rat alveolar macrophages [59]. Beractant contains only two hydrophobic low-molecular weight proteins, i.e. SAP-B and SAP-C. Boston et al. [38] demonstrated that beractant causes a transient increase in [Ca^2+^]_i_ in neutrophils due to G protein-mediated release from intracellular Ca^2+^ stores. Beractant-induced InsP_3_-dependent Ca^2+^ mobilization requires the insertion of monovalent cationic channels by SAP-B and SAP-C, which depolarize the cells by causing Na^+^ influx [38,60]. Likewise, we found that preventing membrane depolarization by exposing NHLF to an external solution devoid of Na^+^, blocked beractant-induced elevation in [Ca^2+^]_i_. The mechanistic link between the positive shift in membrane potential and PLCβ activation is likely to be provided by voltage-dependent GPCRs, such as P2Y1, 5HT2A, thromboxane (TPα), M1 and M3 receptors [61,62]. For instance, cell depolarization activates P2Y1 receptors in rodent megakaryocytes, thereby leading to InsP_3_ synthesis and InsP_3_-dependent Ca^2+^ mobilization, while voltage fails to stimulate TKRs [61,62]. These metabotropic receptors are expressed by human fibroblasts [63] and could mediate the effect of membrane depolarization on PLCβ. Conversely, two abundant phospholipid components of beractant, such as DPPC and DG, do not change intracellular Ca^2+^ levels, just like albumin, which is not contained either in natural surfactant or in beractant.

After lung injury, recovery depends on reestablishment of the air-lung interface through the elimination of intra-alveolar mesenchymal cells. Beractant induces apoptosis and decreases collagen accumulation in NHLF [11]; accordingly, it can be speculated that the use of exogenous surfactant may have a beneficial role in avoiding the formation of intraluminal fibrosis, and that the changes in the intracellular Ca^2+^ concentration observed may be implicated in this effect. A prolonged elevation in [Ca^2+^]_i_, such as that produced by a sustained plateau, is amid the most powerful apoptogenic signals [64]. Accordingly, we found that inhibiting SOCE with La^3+^ and 2-APB prevented beractant-induced NHLF apoptosis. On the other hand, intracellular Ca^2+^ oscillations encode the information driving the Ca^2+^-dependent activation of several transcription factors [34,65]. Intriguingly, Gd^3+^ prevented beractant from suppressing the downregulation of α_1_(I) procollagen transcript. 2-APB reduced α_1_(I) procollagen expression *per se* and could not be used further, but we should recall that this drug is far less selective than 10 μM Gd^3+^ and interferes also with other Ca^2+^-permeable pathways [33]. The hypothesis that the distinct modes of Ca^2+^ signaling induced by beractant in NHLF control different cellular processes is currently under evaluation.

In summary, our results describe for the first time the pattern of Ca^2+^ signals elicited by a natural lung surfactant extract in primary cultures of human lung fibroblasts. The Ca^2+^ response to beractant is triggered by PLCβ recruitment following the activation of a GPCR. The subsequent cleavage of the membrane phospholipid, PIP_2_, leads to the generation of IP_3_, which releases intraluminally stored Ca^2+^, thereby activating SOCE. The interplay between IP_3_-dependent Ca^2+^ mobilization and SOCE results in a variety of Ca^2+^ signals depending on the resting Ca^2+^ levels. Beractant-induced Ca^2+^ signals might protect against pulmonary structural remodeling in IPF as well as other severe fibrosing respiratory diseases.

## Supporting Information

S1 Fig ATP-induced Ca^2+^ signals in NHLF. **A)** Ca^2+^ response to ATP (100 μM) in NHLF. ATP-induced Ca^2+^ elevation was abrogated by **B)** depletion of intracellular Ca^2+^ stores with CPA (10 μM) in absence of extracellular Ca^2+^ (0Ca^2+^), and **C)** by blockage of PLC activity with U73122 (10 μM).
(TIF)

S2 Fig Effect of BTP-2 on the resting [Ca^2+^]_i_ in NHLF. Ca^2+^ signal evoked by the pyrazole derivative, BTP-2 (20 μM), in a single NHLF cell.
(TIF)
