# Supplementary figures and images for: Lung Beractant Increases Free Cytosolic Levels of Ca2+ in Human Lung Fibroblasts
Source: PLoS One. 2015 Jul 31;10(7):e0134564. doi: 10.1371/journal.pone.0134564 (PMC4521834; doi:10.1371/journal.pone.0134564)

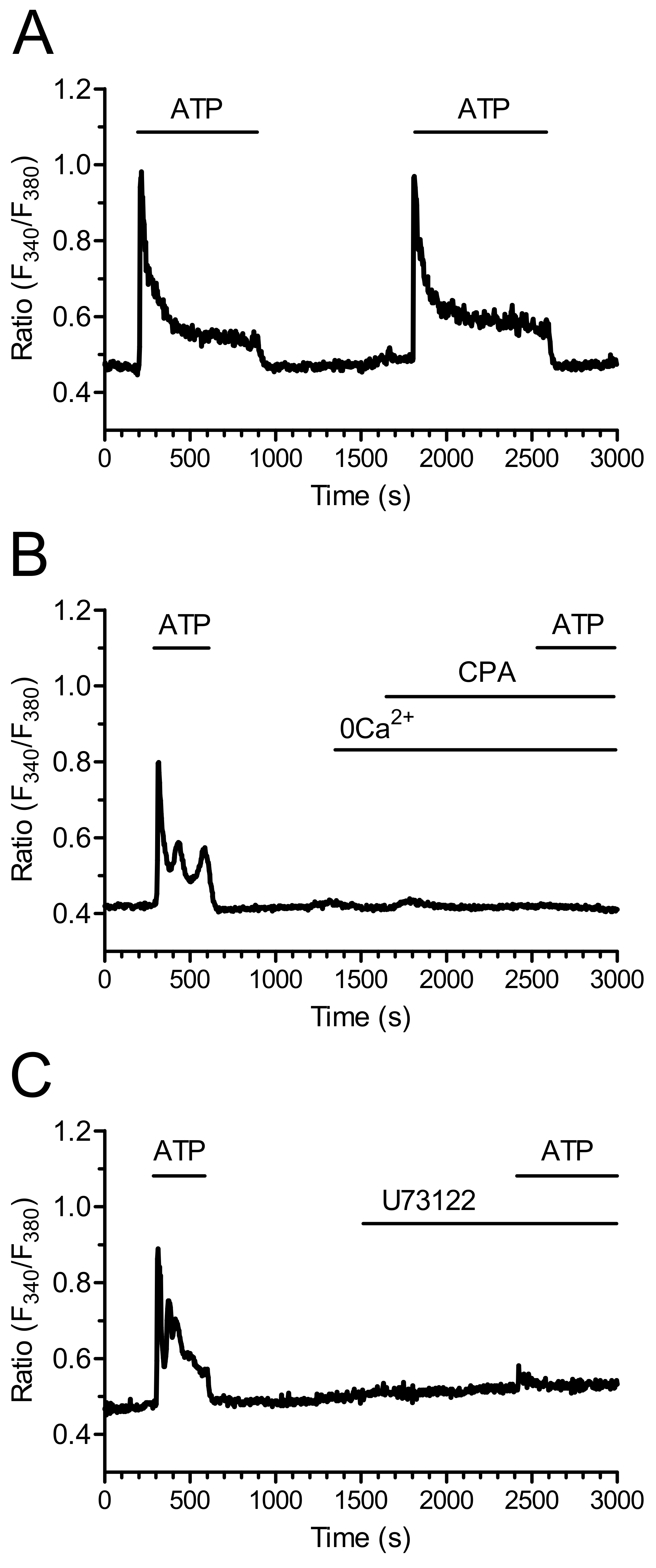

Supplement: S1 Fig — A) Ca2+ response to ATP (100 μM) in NHLF. ATP-induced Ca2+ elevation was abrogated by B) depletion of intracellular Ca2+ stores with CPA (10 μM) in absence of extracellular Ca2+ (0Ca2+), and C) by blockage of PLC activity with U73122 (10 μM). (TIF) [file pone.0134564.s001.tif]

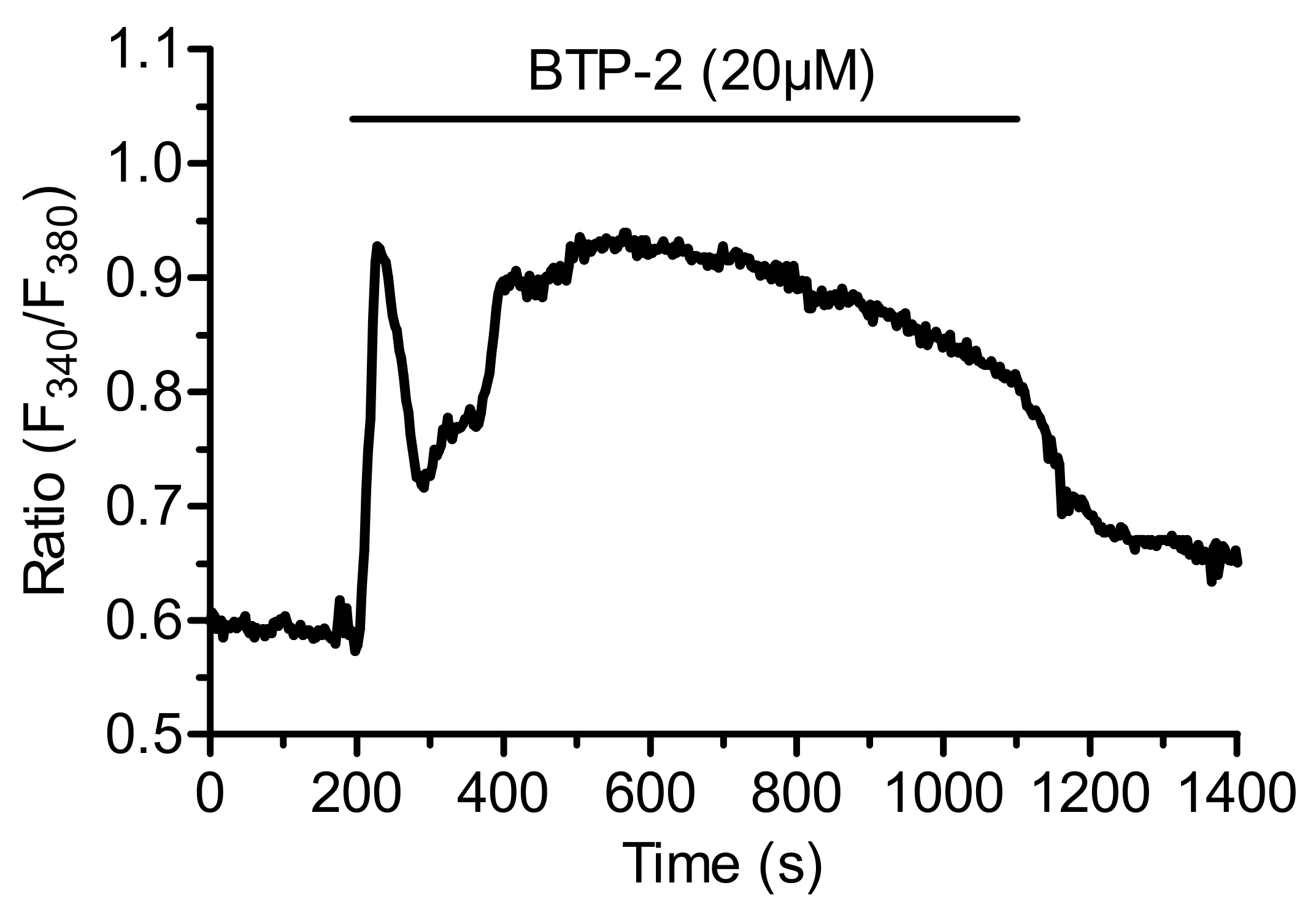

Supplement: S2 Fig — Ca2+ signal evoked by the pyrazole derivative, BTP-2 (20 μM), in a single NHLF cell. (TIF) [file pone.0134564.s002.tif]
